# Halofuginone inhibits tumor migration and invasion by affecting cancer-associated fibroblasts in oral squamous cell carcinoma

**DOI:** 10.3389/fphar.2022.1056337

**Published:** 2022-11-23

**Authors:** Danni Wang, Mei Tian, Yong Fu, Yawei Sun, Liang Ding, Xiaoxin Zhang, Yue Jing, Guowen Sun, Yanhong Ni, Yuxian Song

**Affiliations:** ^1^ Central Laboratory of Stomatology, Nanjing Stomatological Hospital, Medical School of Nanjing University, Nanjing, China; ^2^ Department of Oral and Maxillofacial Surgery, Nanjing Stomatological Hospital, Medical School of Nanjing University, Nanjing, China

**Keywords:** cancer-associated fibroblasts, halofuginone, tumor migration, tumor invasion, oral squamous cell carcinoma (OSCC)

## Abstract

Oral squamous cell carcinoma (OSCC) is the most common malignant tumor in the oral and maxillofacial regions, with a high rate of metastasis. Cancer-associated fibroblasts (CAFs) play critical roles in tumor growth, metastasis and invasion, making them attractive therapeutic targets for cancer treatment. As an old anti-coccidiosis drug for poultry, Halofuginone (HF) has also been reported to possess anti-fibrosis and anti-cancer activities in the recent decades. However, whether it works by targeting CAFs in OSCC, and the mechanisms involved remain unclear. In the present study, we observed HF dose-dependently inhibits OSCC-derived CAF viability and proliferation. Meanwhile, HF decreased the expressions of α-SMA, FSP-1 and PDGFRβ, markers of the malignant phenotype of CAFs, both at mRNA and protein levels. Furthermore, functional studies demonstrated that HF dramatically attenuates the promotion effect of CAFs on OSCC cell migration and invasion. Mechanistically, the inhibition of MMP2 secretion and the upstream TGF-β/Smad2/3 signaling pathway played an important role in these processes. In the orthotopic transplanted tongue carcinoma in mice model, we confirmed that HF administration inhibited tumor growth and lymph node metastasis (LNM) with reduced CAF population, MMP2 expression and collagen deposition in tumor. Altogether, these results indicate that HF can inhibit the migration and invasion of OSCC by targeting CAFs, which will provide new ideas for the treatment of OSCC.

## Introduction

Oral squamous cell carcinoma (OSCC) is the most common malignant tumor in the oral and maxillofacial regions, accounting for approximately 90% of oral malignant tumors (Siegel et al., 2021). At present, clinical treatment of oral squamous cell carcinomas is primarily surgery, and in some cases, combined with chemoradiation or biological therapy. Although the technical level is unceasingly enhanced, the patient’s 5-year survival rate does not improve significantly and remains at approximately 60% (Chen S. H. et al., 2021; Mesia et al., 2021). Therefore, further exploration of effective neoadjuvant is urgently needed.

Emerging evidence has demonstrated that the tumor microenvironment (TME) plays a pivotal role in tumor growth, metastasis and invasion and has a profound impact on the therapeutic effect of tumors (Qin et al., 2021). As a principal constituent of the tumor stroma, Cancer-associated fibroblasts (CAFs) play critical roles in the TME (Kalluri, 2016; Ping et al., 2021).

CAFs affect tumor development in a variety of ways, including maintenance of extracellular matrix, angiogenesis, regulation of tumor metabolism, inhibition of antitumor immunity and promotion of chemotherapy resistance (Zhang et al., 2020) (Dumont et al., 2013). Therefore, CAFs as new therapeutic targets for cancer treatment are attracting increasing attention. In OSCC, our studies and others have demonstrated that the density of CAFs is positively correlated with lymph node metastasis, local recurrence and distant metastasis (Li et al., 2015; Wang et al., 2019). Thus, the role of CAFs in promoting the metastasis and invasion of oral squamous cell carcinoma deserves special attention. Therefore, we considered whether a drug could be used to inhibit the interaction between CAFs and tumors to seek a new adjuvant therapy for oral squamous cell carcinoma.

Halofuginone (HF), an alkaloid originally isolated from the Chinese plant Dichroa febrifuga, has been approved by the US FDA in the 1980s for the prevention of coccidiosis in poultry (Daugschies et al., 1998). In recent years, studies have found that HF can inhibit collagen synthesis, inhibit tissue fibrosis and promote wound healing through the TGF-β/Smad2/3 signaling pathway (Luo et al., 2018; Marty et al., 2021). On the other hand, accumulating evidence has suggested HF can affect tumor cell proliferation, apoptosis and metastasis in many kinds of solid tumors, including lung cancer (Demiroglu-Zergeroglu et al., 2020), rectal cancer (Wang C. et al., 2020), breast cancer (Jin et al., 2014) and esophageal squamous cell carcinoma (Wang Y. et al., 2020). A recent study has demonstrated that HF inhibits the progression of pancreatic ductal carcinoma by inhibiting fibrosis (Elahi-Gedwillo et al., 2019). Since fibroblast is the essential initiator of fibrosis, it seems logical to envisage that HF might regulate CAF activities to change the interactions between CAFs and tumor cells so that enhance its anticancer effect. However, whether it works on CAFs in the TME, especially in OSCC, and the mechanism involved remains largely unknown. The present study aims to fill this gap of knowledge by investigating the effects of HF on OSCC derived CAFs and actions of HF-pretreated CAFs on OSCC cells, which will provide new ideas for HF treatment of OSCC.

## Materials and methods

### Reagents

Halofuginone (Sigma, #64924-67-0, molecular weight: 495.59 g/mol) was dissolved in phosphate buffer solution (PBS) at pH 5.3 at a concentration of 1 mM as a stock solution and diluted to the indicated concentrations with medium before each test.

### Cell culture and preparation of conditioned medium

CAFs and NFs (normal fibroblasts) were isolated from the tumor tissues and the adjacent normal tissues of OSCC patients treated at the Nanjing Stomatological Hospital, Medical School of Nanjing University as previously described (Ding et al., 2018; Wang et al., 2019). The study was approved by the ethics committee for clinical study of Nanjing Stomatological Hospital. CAFs and NFs were cultured in fibroblast medium (Cat No. 2301, SclenCell™). For further experiments, CAFs were used between the third and seventh passage. OSCC cell lines HSC3 and HN6 were cultured in complete DMEM medium supplemented with 10% FBS, penicillin (100 U/mL) and streptomycin (0.1 mg/ml).

Conditioned medium (CM) from CAFs was prepared as follow: CAFs were seeded into four dishes, treated with different concentrations of HF (25, 50, 100 nM) or vehicle (PBS, pH = 5.3) for 6 h, the cells were then washed twice with PBS, and cultured in fresh complete media for another 48 h. Cell fragments were removed from the supernatants by centrifugation at 3,000 rpm for 10 min at 4°C. Supernatants were collected and used immediately or stored at −20°C for later use.

### Western blotting

Proteins from cells were extracted in RIPA lysis buffer with a mixture of protease and phosphatase inhibitors on ice. The protein concentration of each sample was determined using a BCA protein assay kit (Vazyme, China). Total protein (20 μg) was separated by 4%–20% sodium dodecyl sulfate–polyacrylamide gel electrophoresis and was transferred to polyvinylidene difluoride membrane. Membranes were blocked at room temperature for 1 h with 5% BSA in Tris-buffered saline, and incubated with primary antibodies at 4°C overnight. After washing, the membranes were incubated at room temperature with HRP-conjugated secondary antibody for 1 h, protein bands were detected by a protein imaging system (Tanon, China). The primary antibodies were as follow: α-SMA (#ab5694) was obtained from the Abcam company. FSP-1 (# 66489), Vimentin (#10366), PDGFRβ (#13449) and MMP2 (#10373) were obtained from Proteintech company. p-Smad2/3 (#8828S), p-AKT (#4060S), p-JNK (#9255S), p-P38 (#4511S), Anti-rabbit IgG, HRP-linked Antibody (#7074), Anti-mouse IgG and HRP-linked Antibody (#7076) were obtained from Cell Signaling Technology.

### Immunofluorescence

CAFs were cultured in confocal dishes and treated with HF or not as indicated. Cells were then fixed with 4% paraformaldehyde and permeated with 1%Trition X-100 solution at room temperature. Following three extensive washings with PBS, the samples were blocked with 5% BSA and incubated with primary antibodies at 4°C overnight. After rinsing three times in PBS, the samples were incubated with different secondary antibodies at a 1:400 dilution for 1.5 h at room temperature in the dark, and then the nuclei were stained with DAPI. Samples were visualized using Nikon A1 confocal microscope (Nikon, Japan).

### Cell viability assay

CAFs were seeded into 96-well plate (4000 cells per well, in 100 µL) overnight and treated with different concentrations of HF (25, 50, 100, 200, 400 nM) or vehicle for 24 h or 48 h. HSC3 or NH6 cells were seeded into 96-well plate (5000 cells per well, in 100 µL) overnight, and cultured in different treated CM from CAFs for 48 h. Cell counting kit-8 (CCK8) (Dojindo, Japan) was used according to the manufacturer’s instruction to detect cell viabilities. Absorbance of 450 nm was measured by a microplate reader Spectra Max M3 (Molecular Devices, United States).

### Apoptosis assay

CAFs were seeded into 12-well plate (2 × 10⁵ cells per well) and treated with HF (25, 50, 100 nM) or vehicle for 48 h. To analyze the effect of HF on cell-apoptosis, the CAFs were digested by trypsin (excluding EDTA) and centrifuged at 1,300 rpm, 4°C for 5 min. After washing with PBS, the cells were stained with Annexin-V-FITC/PI (Vazyme, China) according to the manufacturer’s instruction. Flow cytometry was performed using FACSCalibur flow cytometer (BD Bioscience, San Diego), and data were analyzed with FlowJo software (Treestar, Inc., San Carlos).

### EdU assay

CAFs were plated to 12-well plate and treated with HF (25, 50, 100 nM) or vehicle for 48 h. EdU staining proliferation kit was used according to the manufacturer’s instruction (Beyotime, China). Briefly, cells in plates were added with EdU solution and incubated for 2 h and then treated with 4% formaldehyde. After fixation, permeable solution was added and incubated for 10–15 min. After cleaning, 500 μL Click reaction solution was added to each well and incubated at room temperature in dark for 30 min. Cells were observed and photographed under a fluorescence microscope.

### Quantitative real-time polymerase chain reaction (qRT-PCR)

After HF-treated CAFs were cultured for 48 h, RNA of CAFs was extracted. RNA was obtained using Trizol reagent following the manufacturer’s procedure and then reverse transcribed into cDNA using HiScript III RT SuperMix (Vazyme, China). Subsequently, real-time quantitative PCR was performed using the AceQ^®^ qPCR SYBR^®^ Green Master Mix (Vazyme, China) and Vii7 Real-Time PCR System (Applied Biosystems, CA). The primer sequences used in this study were obtained from commercial sources and are displayed in Table 1.

**TABLE 1:** Primers used for real-time quantitative PCR analysis.

Gene	Forward primer	Reverse primer
a-SMA	CCT​GTG​TTG​TGG​TTT​ACA​CTG​G	GGG​GGA​ATT​ATC​TTT​CCT​GGT​CC
FSP-1	GAT​GAG​CAA​CTT​GGA​CAG​CAA	CTG​GGC​TGC​TTA​TCT​GGG​AAG
PDGFRβ	AGC​ACC​TTC​GTT​CTG​ACC​TG	TAT​TCT​CCC​GTG​TCT​AGC​CCA
N-Cadherin	TCA​GGC​GTC​TGT​AGA​GGC​TT	ATG​CAC​ATC​CTT​CGA​TAA​GAC​TG
Vimentin	GAC​GCC​ATC​AAC​ACC​GAG​TT	CTT​TGT​CGT​TGG​TTA​GCT​GGT
GAPDH	GGA​GCG​AGA​TCC​CTC​CAA​AA	GGC​TGT​TGT​CAT​ACT​TCT​CAT​GG

### Wound healing assay

To study the effects of HF on cell migration *in vitro*, a wound healing assay was performed using HSC3 and NH6 cells. Cells were seeded in 6-well plate and grown until the cell confluence reached 90%. Then cells were serum-starved for 12 h. A linear wound was created in the confluent monolayer using a 200-µL pipette tip. After PBS washing, different concentrations of CM were added and mixed 1:1 with normal serum-free media. The trace widths of different treatment groups were observed at 0 h, 8 h, 16 h, and 24 h and photographed and recorded. The time points with the largest differences were taken for results analysis.

### Migration and invasion assay

For migration assay, HSC3 or NH6 cells (1×10^5^ cells per well) were seeded in the upper chambers of 24-well Transwell plate (with 8 µm pore size, Costar) and starved overnight. CAFs were seeded into another 24-well plate (5 × 10⁴ cells per well) and treated with HF (25, 50, 100 nM) or vehicle for 6 h. CAFs were then washed twice with PBS and replaced with fresh complete media. Tumor cells in the Transwell inserts were carefully transferred to the 24-well plate paved with CAFs, and co-cultured for 12 h. For invasion assay, matrigel was spread on the Transwell chamber and co-cultured for 24 h. After 12 h or 24 h of co-culture, tumor cells were fixed with 4% paraformaldehyde, stained with crystal violet, and observed and counted under a microscope.

### Xenograft tumor models

Female Balb/c nude mice (4–6 weeks) were kept in standard environment with the unified breeding. All the experiments were approved by the Animal Research Committee of Medical College Affiliated to Nanjing University and conformed to the ethical standards with the guidelines of the National Animal Care and Ethics Institution. A total of 1×10^6^ CAFs and 2×10^5^ HN6 cells (CAFs and HN6 were mixed at a ratio of 5:1) were injected on the right tongue edge of each mouse to observe tumor formation. After 14 days, 10 mice with successfully established tongue tumor were randomly divided into two groups: the control group (n = 5) and the HF treatment group (n = 5). The treatment group was intraperitoneally injected with 0.5 mg/kg HF every other day for 14 days, and the control group injected with PBS (pH = 5.3). On the 28th day, the mice were sacrificed by cervical dislocation under general anesthesia, and the tongues and cervical lymph nodes were removed. Tissues were soaked overnight in 4% paraformaldehyde, dehydrated conventionally and embedded in paraffin.

### Masson staining

Paraffin embedded tumor tissues of mice were cut into 5 μm slices for Masson staining. The distribution of collagen in tumor tissue was observed and photographed under a cell imaging system, and the staining results were analyzed by using ImageJ software.

### Immunohistochemistry analysis

Paraffin embedded tumor tissues of mice were cut into 5 μm slices, and the dehydration was reached with the different concentrations of ethanol after being dewaxed in xylene. The sections were subjected to antigen retrieval using 10 mM citrate buffer (92°C for 30 min). Hydrogen peroxide (3%) and BSA (5%) were used to block endogenous peroxidase activity and nonspecific staining for 10 min and 20 min, respectively. Immunohistochemical staining for α-SMA and MMP2 was performed. Protein levels of α-SMA and MMP2 were evaluated according to staining intensity and the percentage of positive cells.

### Statistical analysis

All results were expressed as the mean ± S.E.M unless otherwise indicated. Each experiment was repeated at least three times. Data with *p* < 0.05 were considered statistically significant. Analysis of experimental results was performed using GraphPad Prism (GraphPad Software v8.0).

## Results

### Extraction and phenotypic identification of primary cancer-associated fibroblasts

**FIGURE 1:**
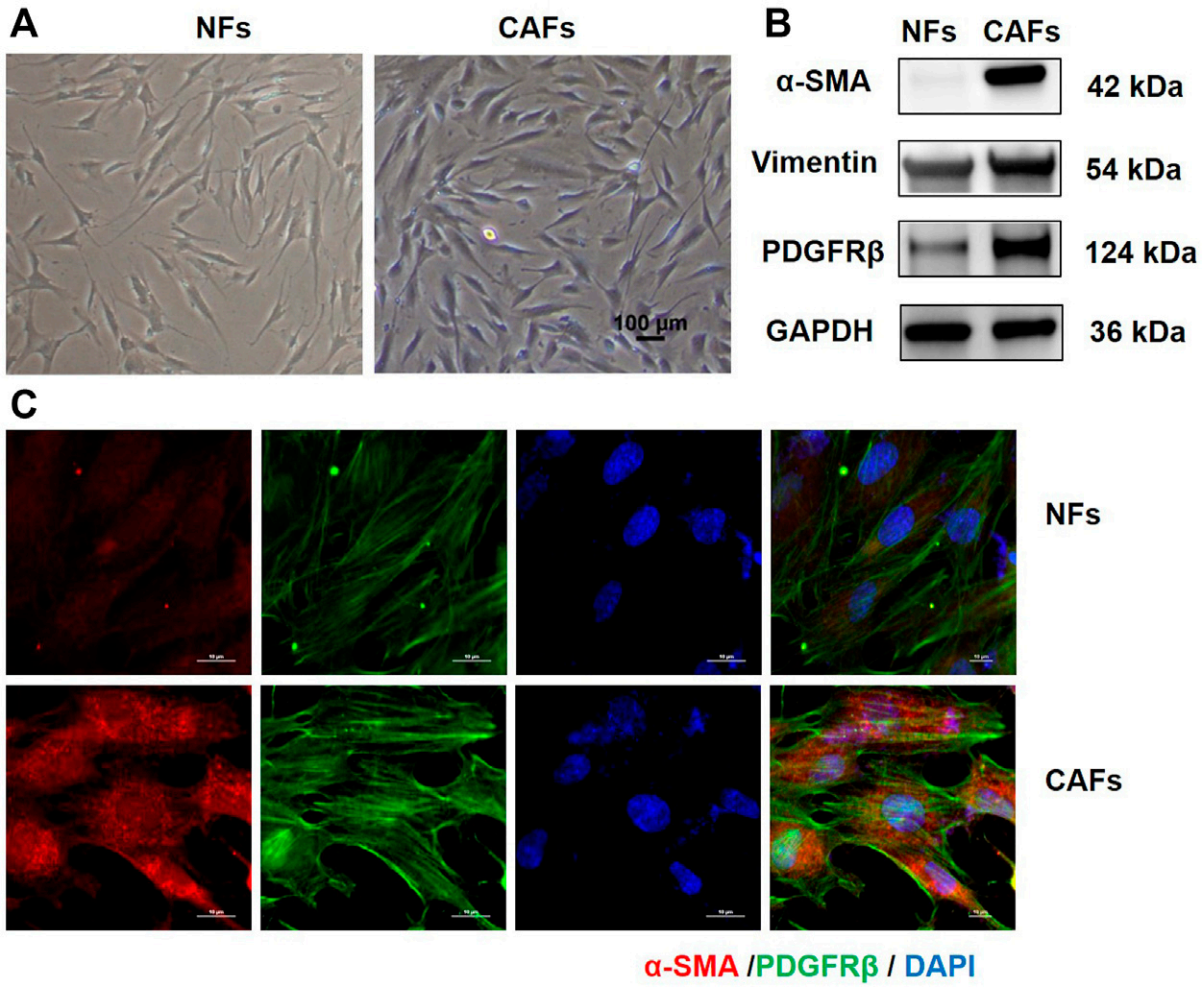
Culture and identification of primary tumor-associated fibroblasts. **(A)** Morphology of NFs and CAFs under invert microscope (100×). **(B)** Protein levels of α-SMA, Vimentin and PDGFRβ in NFs and CAFs detected by Western blotting analysis. **(C)** Expression levels of α-SMA and PDGFRβ in NFs and CAFs were observed by immunofluorescence.

We first extracted primary CAFs and NFs from the tumor tissue and the adjacent normal tissue of OSCC patients. The morphologies of CAFs and NFs were observed under invert microscope. As shown in Figure 1A, both CAFs and NFs have large and long spindle shapes. Phenotypic identifications of CAFs were then conducted. Western blotting analysis showed that, compared with NFs, CAFs expressed higher level of mesenchymal markers, such as α-SMA, Vimentin and PDGFRβ (Figure 1B). Results of immunofluorescence also showed that CAFs express higher levels of α-SMA and PDGFRβ (Figure 1C).

### Halofuginone decreased CAF proliferation

**FIGURE 2:**
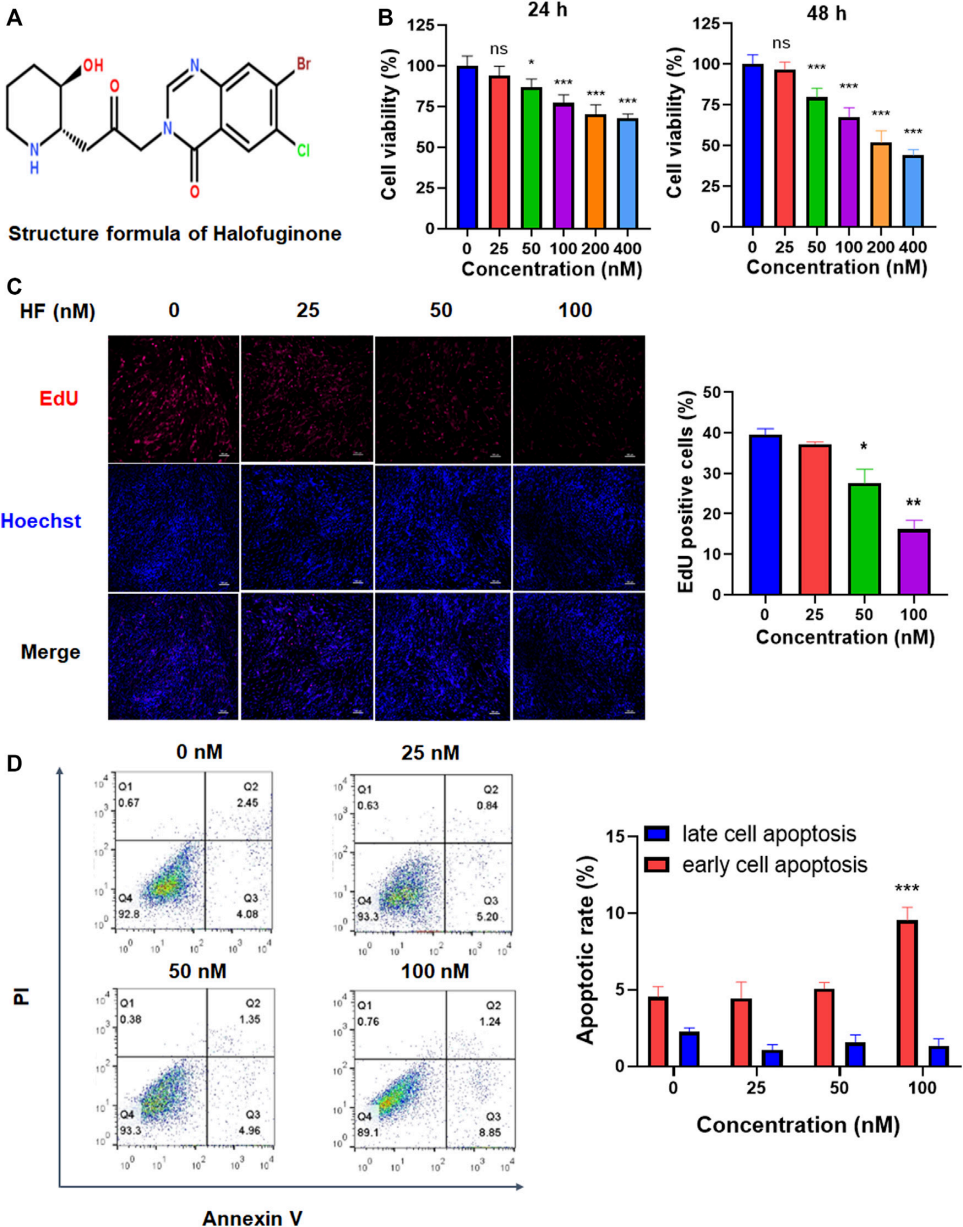
The effects of Halofuginone on CAF viability, proliferation and apoptosis. **(A)** Chemical structure of HF. **(B)** CAFs were treated with or without HF (25, 50, 100, 200, 400 nM) for 24 h or 48 h, and cell viability of CAFs was detected by CCK8; **(C)** CAFs were treated with or without HF (25, 50, 100 nM) for 48 h, cell proliferation was detected by EdU staining. **(D)** Cell apoptosis was analyzed by flow cytometry. *:*p* < 0.05, **:*p* < 0.01.

CAFs were treated with different concentrations of HF (structure was shown in Figure 2B) and their viabilities were tested using the CCK-8 assay. As shown in Figure 2A, HF inhibited CAF cell viability in a dose-dependent manner. Furthermore, EdU assay showed HF inhibited the proliferation of CAFs at 50 and 100 nM (Figure 2C). We further investigated the effect of HF on cell apoptosis. Flow cytometry results showed that only 100 nM HF significantly induced early apoptosis at a ratio of 10% compared with 5% in control group. However, there were no significant differences among low concentration groups and control group either for early or late apoptosis (Figure 2D).

### Halofuginone reduced the expressions of CAF phenotypic markers

**FIGURE 3:**
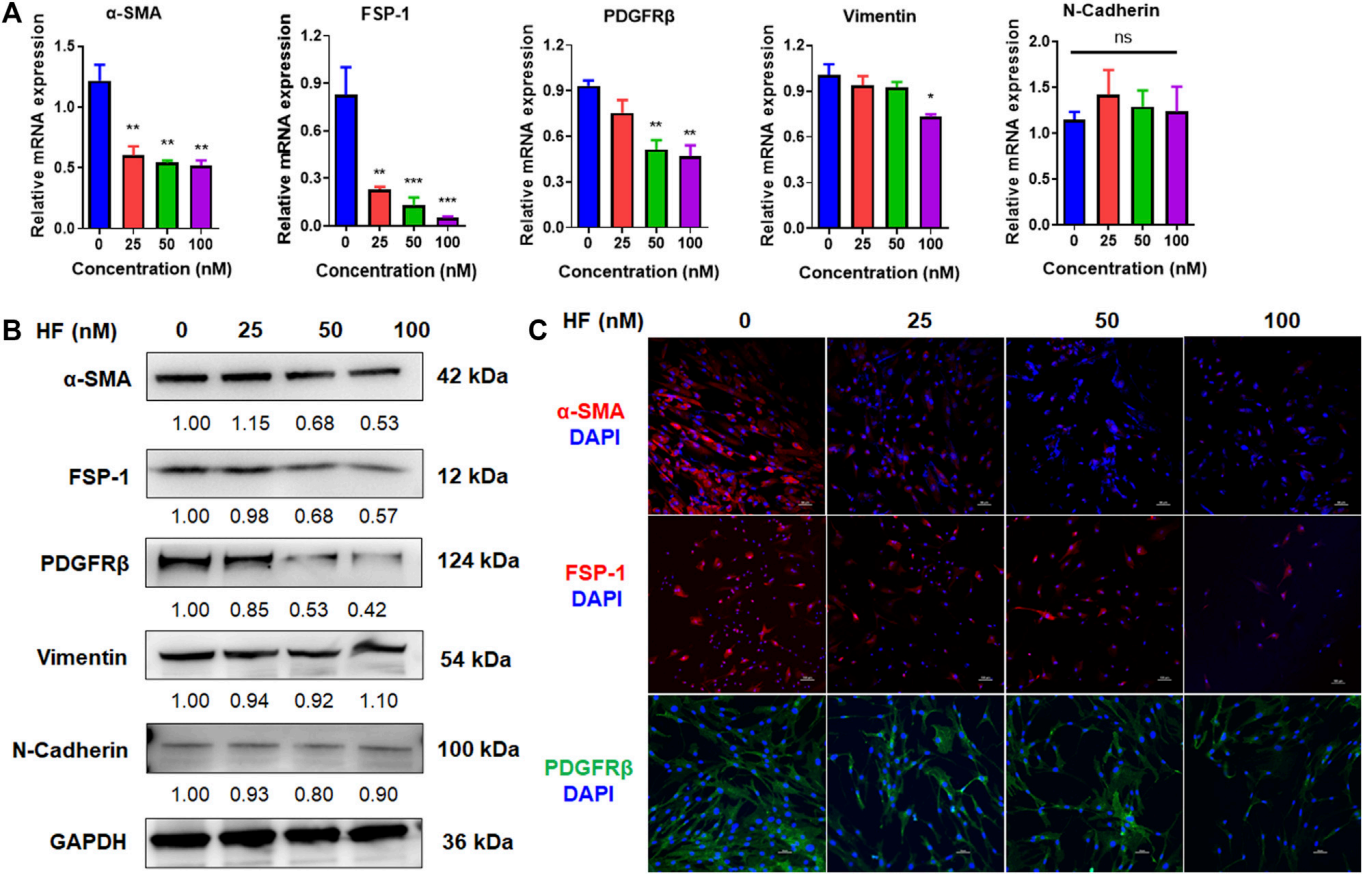
Halofuginone inhibited the expressions of CAF phenotypic markers. CAFs were treated with or without HF (25, 50, 100 nM) for 48 h. The mRNA and protein levels of CAF phenotypic markers α-SMA, FSP-1, PDGFRβ, N-cadherin and vimentin were detected by qRT-PCR **(A)** and Western blotting **(B)**, respectively. **(C)** The Protein levels of α-SMA, FSP-1, PDGFRβ were confirmed by immunofluorescence assays. *:*p* < 0.05, **:*p* < 0.01.

Activated fibroblasts express high levels of α-SMA, FSP-1, PDGFRβ, N-cadherin and vimentin, which are markers of the malignant phenotype of CAFs. To determine whether HF treatment altered CAF phenotype, we treated CAFs with HF (25, 50, 100 nM) for 24 h or 48 h. The mRNA and protein levels of these markers were detected by using qRT-PCR, western blotting and immunofluorescence assay, respectively. We found that the mRNA levels of these markers didn’t change by HF treatment at 24 h (data not shown), while α-SMA, FSP-1, PDGFRβ and vimentin but not N-cadherin decreased significantly at 48 h treatment (Figure 3A). Meanwhile, results of western blotting showed that HF dose-dependently inhibited expression levels of α-SMA, FSP-1 and PDGFRβ but not N-cadherin or vimentin (Figure 3B). The subsequent immunofluorescence assay confirmed these results (Figure 3C).

### HF-pretreated CAFs reduced the migration and invasion of OSCC cells

**FIGURE 4:**
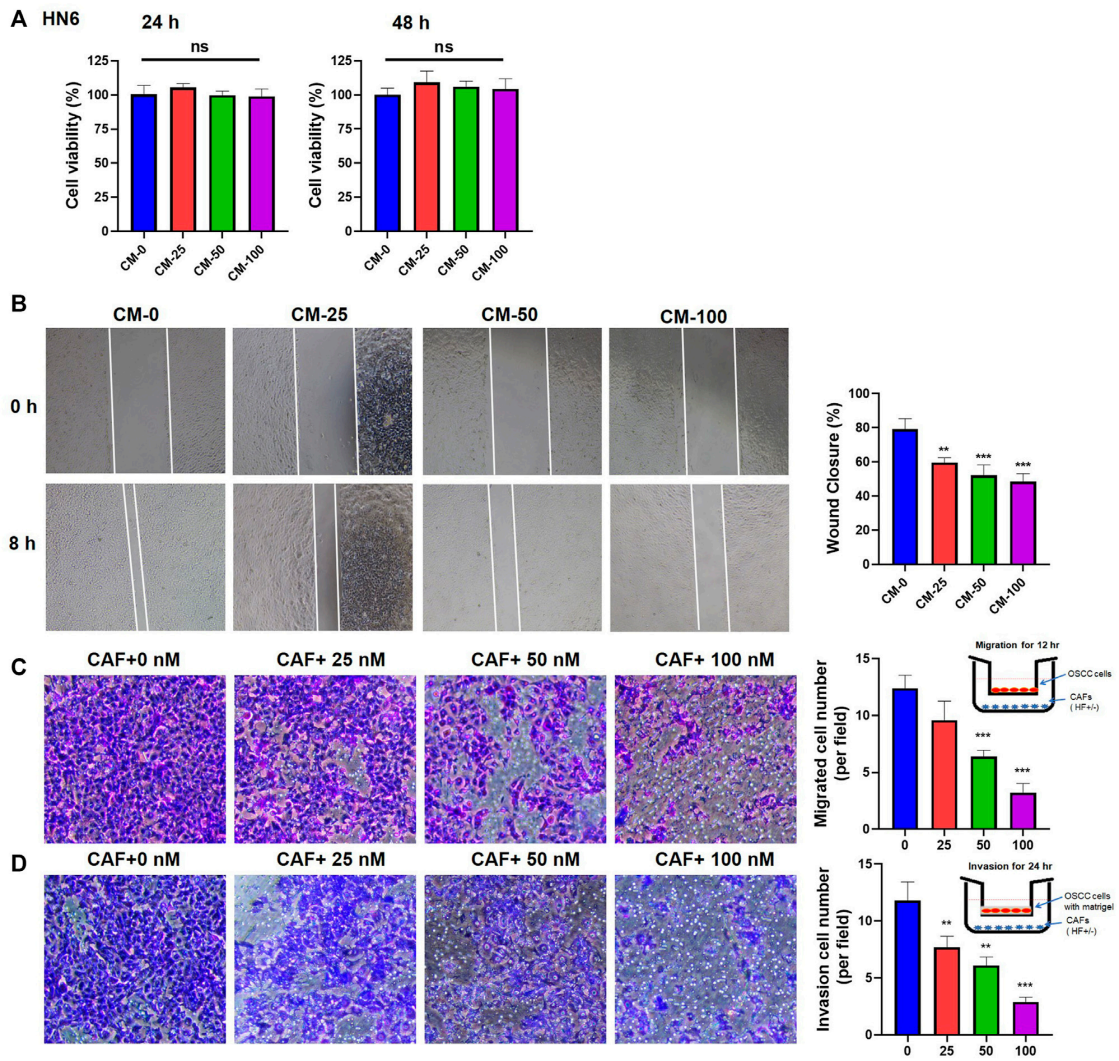
Effects of HF-treated CAFs on the proliferation, migration, and invasion of tumor cells. **(A)** CM had no effect on the proliferation of HN6 cells. **(B)** CM treated with high-concentration (100 nM) HF could significantly inhibit the migration of HN6 cells (100×). Transwell experiments showed that HF-treated CAFs significantly inhibit tumor migration **(C)** and invasion **(D)**. *:*p* < 0.05, **:*p* < 0.01, ***:*p* < 0.001.

To observe whether HF-treated CAFs could affect the proliferation, migration and invasion of tumor cells, CMs from HF-treated CAFs or the cells (in Transwell) were co-cultured with OSCC cells, then the cell viability, migration and invasion activities were detected. Results of CCK-8 assay showed that different CMs had no significant effect on the proliferation of OSCC cell line HN6 (Figure 4A). However, wound healing assay results showed that the CM collected from 100 nM HF-treated CAFs could inhibit the migration ability of HN6 cells after 8 h of treatment (Figure 4B). Further, transwell migration and invasion experiments also showed that HF-treated CAFs could obviously decrease HN6 cell migration (Figure 4C) and invasion in dose-dependent manners (Figure 4D). The same results were observed in HSC3 cell line (Supplemental Figure S1).

### Halofuginone inhibited MMP2 expression and TGF-β/Smad2/3 signaling pathway in CAFs

**FIGURE 5:**
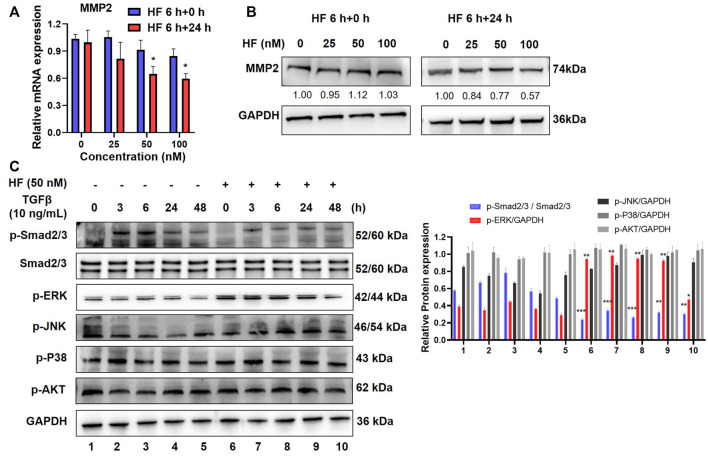
Halofuginone inhibited MMP2 expression and TGF-β/Smad2/3 signaling pathway in CAFs. **(A)** CAFs were treated with or without HF (25, 50, 100 nM) for 6 h and replaced with complete media and cultured for further 24 h. The mRNA level of MMP2 in CAFs was detected by qPCR. **(B)** The protein level of MMP2 was detected by Western bloting, and bands were quantified using ImageJ software. Similar results were seen in at least three independent experiments. *:*p* < 0.05. **(C)** CAFs were treated with HF (50 nM) for 48 h with or without TGFβ (10 ng/ml, R&D) stimulation for 3 h, 6 h, 24 h, and 48 h to stimulate the activation of the signaling pathway. Western blotting was used to detect the changes in phosphorylated Smad2/3, ERK, JNK, p38 and AKT protein levels.

Matrix metalloproteinases 2 (MMP2) is a well-known enzyme synthesized by fibroblasts and controlled ECM degradation and remodeling to influence tumor migration. To investigate whether HF inhibit tumor cell migration and invasion by regulating ECM, we carried out qRT-PCR and western blotting to detect the effect of HF on the expression of MMP2 at the mRNA and protein levels. Results showed that either MMP2 mRNA (Figure 5A) or protein (Figure 5B) level in CAFs was not changed after 6 h treatment of HF, but decreased 24 h later after continuous culture in complete media. TGF-β is commonly high expressed in tumor tissues of OSCC patients and extensively associated with cell migration and tumor metastasis (Lu et al., 2019). Several studies suggest TGF-β increases the synthesis of MMP2 and enhances its activity in tumor cells (Kim et al., 2021). Thus, we tested whether HF affect the TGF-β signaling pathways, including the SMAD-dependent canonical pathway or SMAD-independent non-canonical MAPK and PI3K/AKT signaling pathways (Chen J. et al., 2021). CAFs were treated with or without 50 nM HF followed by stimulation with 10 ng/ml TGF-β for indicated time points to stimulate the activation of related signaling pathways. Results showed that TGF-β activated the phosphorylation of Smad2/3 but not ERK, JNK, p38 or AKT in CAFs. However, HF treatment decreased the phosphorylation of Smad2/3 and upregulated the phosphorylation of ERK1/2 (Figure 5C). Thus, we hypothesized that HF inhibited the proliferation and activation of CAFs through the TGF-β/Smad2/3 signaling pathway which resulted to inhibit tumor cell migration and invasion.

### Halofuginone inhibited tumor growth and metastasis *in vivo*

**FIGURE 6:**
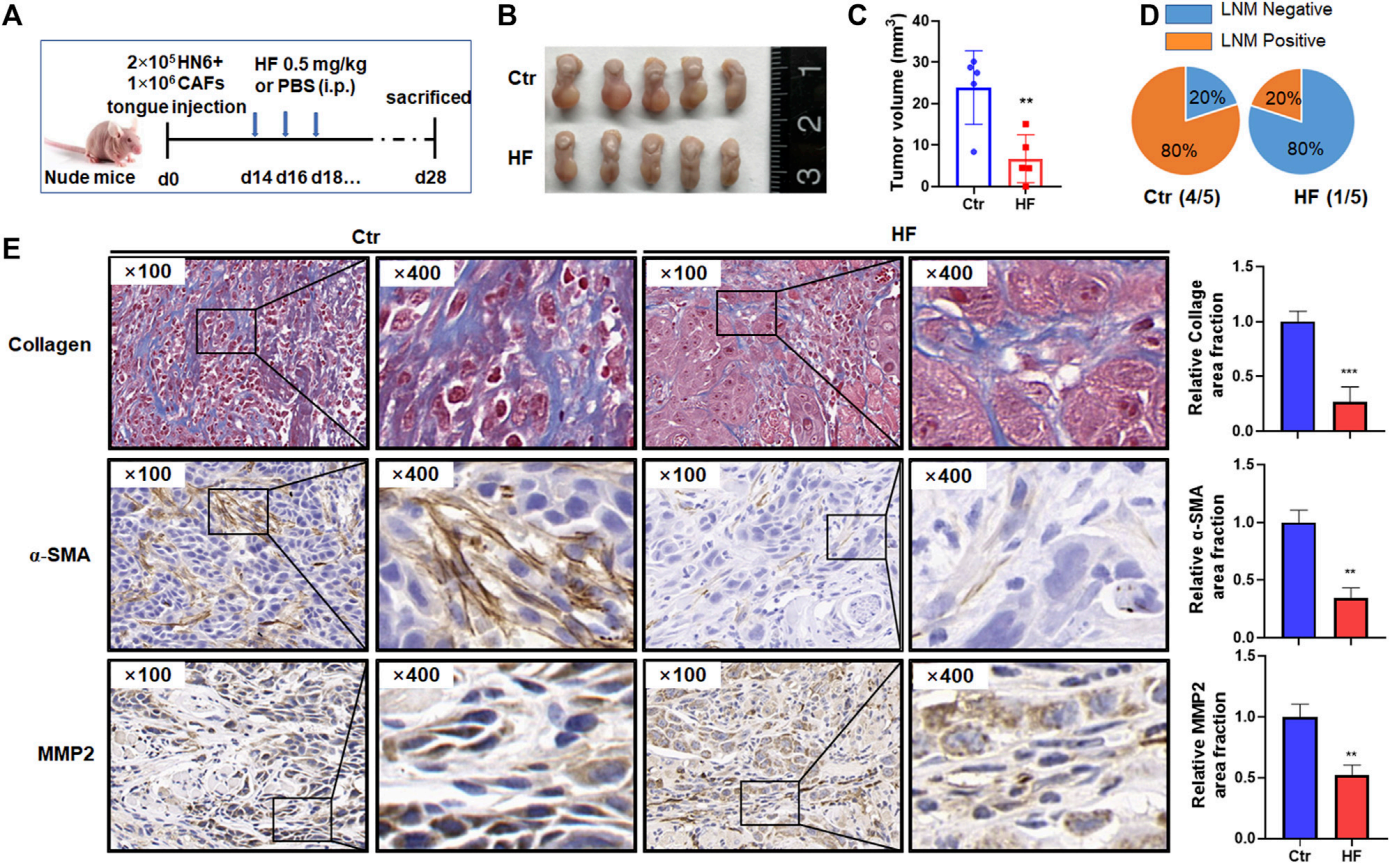
Halofuginone decreased tumor growth, LNM, CAF population, MMP2 expression and collagen deposition *in vivo*. **(A)** Experimental design for the *in vivo* study. Female Balb/c nude mice (4–6 weeks) successfully established tongue tumor were randomly divided into two groups: the control group (PBS, n = 5) and the HF treatment group (n = 5). The treatment group was intraperitoneally injected with 0.5 mg/kg HF every other day for 14 days. Tumor measurement **(B,C)** and LNM percentage **(D)** were made after mice were sacrificed on day 28th. **(E)** Masson staining was conducted to determine the collagen deposition in tumor stroma. Immunohistochemistry was used to evaluate the α-SMA and MMP2 protein levels. **:*p* < 0.01, ***:*p* < 0.001.

To verify the above findings *in vivo*, we established an orthotopic model of OSCC in nude mice by injecting HN6 and CAFs into the tongue (Figure 6A). As shown in Figure 6B, HF administration dramatically inhibited tumor growth in the tongue (Figure 6B). The mean volume of tumors at day 28 in the mice who received HF (23.93 ± 8.91 mm^3^) was significantly decreased compared with control mice (6.69 ± 5.76 mm^3^) (*p* < 0.01, Figure 6C). In addition, the percentage of lymph node metastasis (LNM) was also remarkably reduced in HF group (20% LNM^+^) compared with control group (80% LNM^+^) (Figure 6D). We further performed Masson staining and immunohistochemical staining on tumor tissues. Results showed that HF treatment decreased collagen deposition in tumor stroma, and the expression of α-SMA and MMP2 were also reduced (Figure 6E).

## Discussion

Halofuginone is an alkaloid derived from quinazolinone with a low molecular weight that has a significant antifibrotic effect and can inhibit the production of type I collagen and reverse the fibrosis state (McGaha et al., 2002). Accumulating evidences suggest that halofuginone can induce apoptosis of several kinds of cancer cells and inhibit migration of cancer cells in some ways. Recent studies have also found that HF can inhibit the progression of pancreatic ductal carcinoma by inhibiting CAF activity and breaking the barrier affecting targeted drug delivery (Elahi-Gedwillo et al., 2019). Here, our results demonstrated for the first time that halofuginone dose-dependently inhibits OSCC-derived CAF viability and proliferation. Meanwhile, HF decreases the expressions of α-SMA, FSP-1 and PDGFRβ, markers of the malignant phenotype of CAFs, both at mRNA and protein levels. It seems that HF can partially shift CAFs to a less active phenotype, somewhat like the NF phenotype.

It is widely accepted that CAFs are crucial in the occurrence, development, invasion and metastasis of OSCC (Erdogan and Webb, 2017; Haga et al., 2021). Therefore, it is important to explore whether HF could inhibit the migration and invasion of OSCC cells by affecting CAFs. Our further experiments confirm this hypothesis that HF dramatically attenuates the promotion effect of CAFs on OSCC cell migration and invasion. Furthermore, we have also shown the beneficial effects of HF in the orthotopic transplanted tongue carcinoma mouse mode. We demonstrate that HF can inhibit tumor growth, LNM, collagen deposition and the population of α-SMA positive CAFs, indicating that HF treatment can reduce the proliferation, activation and tumor invasion of CAFs in the tumor region. Although the direct effect of HF on OSCC cells has not been reported yet, and not showed in the present study, we believe that HF can decrease the malignancy of OSCC not only by directly targeting tumor cells but also by targeting CAFs in the tumor microenvironment. Therefore, HF is a suitable candidate for anticancer combination therapy and tumor metastasis inhibition.

When exploring the mechanism of HF effects on CAFs, we find HF inhibits the secretion of MMP2 and the upstream TGF-β/Smad2/3 signaling pathway but active the ERK pathway. In fact, the signaling pathway by which HF affecting on remains controversial. Roffe et al. (2010) found that HF inhibited Smad3 phosphorylation in muscle cells was due, at least in part, to HF-dependent activation of ERK, JNK and p38. Zeng et al. (2017) suggested that HF treatment robustly suppressed the TNF-α-induced phosphorylation of p38 and JNK, but didn’t effluence ERK activation in fibroblast-like synoviocytes. However, Li et al. (2021) demonstrated that HF inhibited cancer cell proliferation by downregulating ERK phosphorylation in lung cancer cells. Thus, the effect of HF on ERK phosphorylation in different environments is not the same. Our results demonstrated that HF inhibits the proliferation activity of CAFs and the expression of malignant phenotypic markers through the TGF-β/Smad2/3 signaling pathway. Meanwhile, HF upregulated the phosphorylation of ERK1/2 in CAFs.

Taken together, we believe that HF can inhibit the migration and invasion of OSCC by acting on CAFs. Our results will provide new ideas for HF treatment of postoperative recurrence and metastasis of OSCC.

## Data Availability

The original contributions presented in the study are included in the article/Supplementary Material, further inquiries can be directed to the corresponding authors.

## Supplementary material

SUPPLEMENTARY FIGURE S1 Effects of HF-treated CAFs on the proliferation, migration, and invasion of HSC3 cells. **(A)** CM had no effect on the proliferation of HSC3 cells. **(B)** CM treated with high-concentration (100 nM) HF could significantly inhibit the migration of HSC3 cells (100×). Transwell experiments showed that HF-treated CAFs significantly inhibit tumor migration **(C)** and invasion **(D)**. *:*p* < 0.05, **:*p* < 0.01, ***:*p* < 0.001.
