# Supplementary figures and images for: Halofuginone inhibits tumor migration and invasion by affecting cancer-associated fibroblasts in oral squamous cell carcinoma
Source: Front Pharmacol. 2022 Nov 23;13:1056337. doi: 10.3389/fphar.2022.1056337 (PMC9726898; doi:10.3389/fphar.2022.1056337)

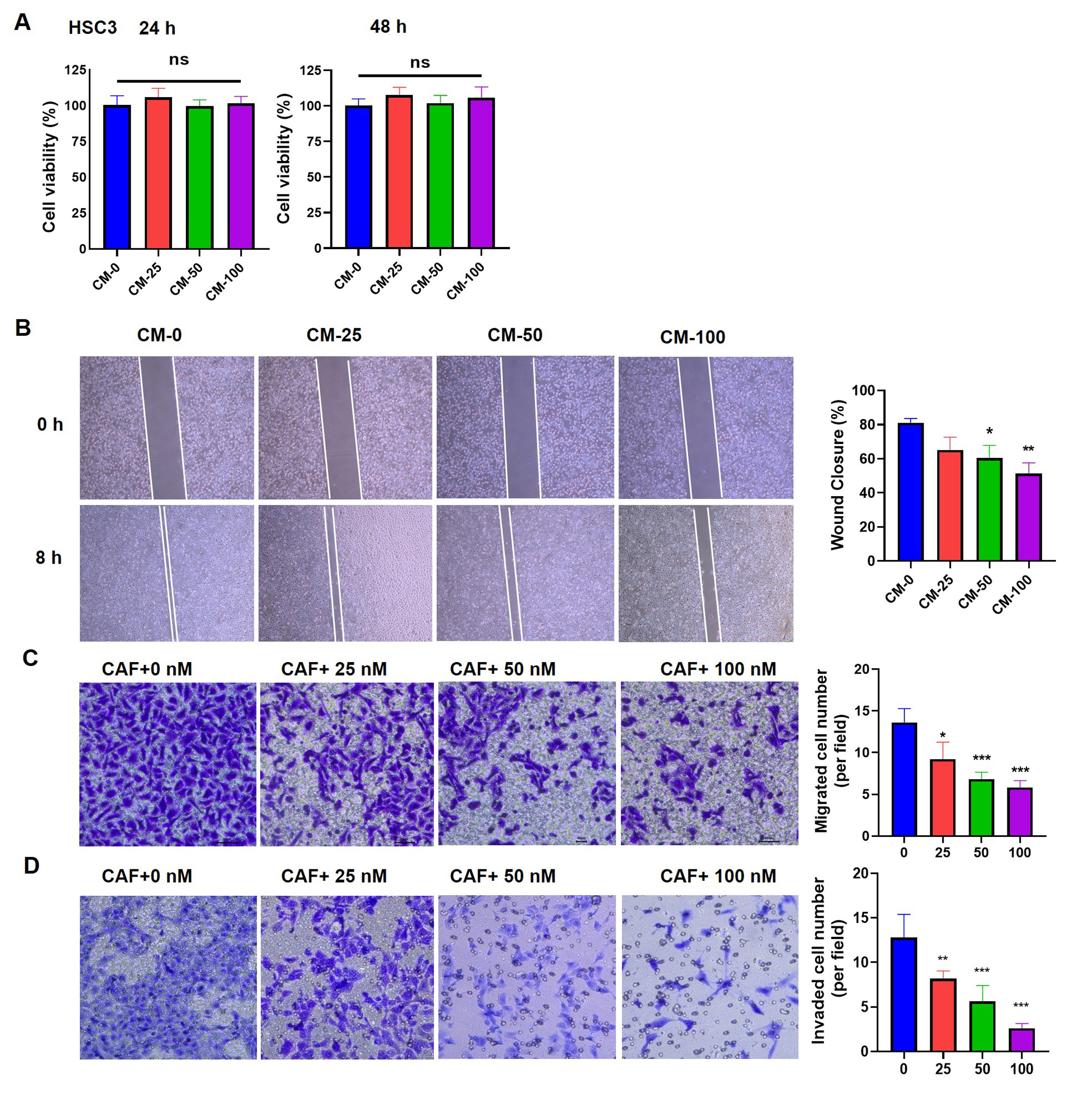

Supplement: Supplementary file 1 [file Image1.jpeg]
